# The Buffer Illusion: How TRIS and HEPES Enhance Bioactivity of Bioglass 45S5

**DOI:** 10.1021/acsomega.5c12487

**Published:** 2026-10-12

**Authors:** Tereza Přívracká, Karolína Opavová, Petr Bezdička, Jan Labuta, Dana Rohanová

**Affiliations:** † Department of Glass and Ceramics, University of Chemistry and Technology Prague, Technická 5, Prague 6 166 28, Czech Republic; ‡ Institute of Inorganic Chemistry of the Czech Academy of Sciences, Husinec-Řež 1001, Husinec-Řež 250 68, Czech Republic; § International Center for Materials Nanoarchitectonics (MANA), National Institute for Materials Science (NIMS), 1-1 Namiki, Tsukuba, Ibaraki 305-0044, Japan

## Abstract

*In vitro* tests of glass, glass-ceramic, or ceramic biomaterials such as synthetic hydroxyapatite (HA) and β-tricalcium phosphate (β-TCP) are performed using simulated body fluid (SBF), in which the pH is maintained using TRIS buffer (ISO standard 23317:2014; recently updated as ISO standard 23317:2025). When evaluating the experiments, we not only looked at the tested material itself but also analyzed the SBF leachate. We have found out that TRIS buffer, as well as other tested buffers (Good’s buffers), actively interacts with glass and ceramic materials. They significantly accelerate their dissolution (up to 3× higher compared to unbuffered SBF). This increases the local supersaturation of biogenic ions near the surface and promotes the formation of the expected hydroxyapatite (the newly formed hydroxyapatite layer is labeled as HAp). In this work, we focused on bioglass 45S5 to observe the corrosion rate of the glass surface on samples in the form of bulk, in SBF solutions buffered with (i) TRIS and (ii) HEPES, and we used (iii) SBF without any buffer for comparison. The glass corrosion rate and the growth kinetics of the new HAp layer (SEM/EDS, XRD, FTIR) were compared with the results of the leachate analyses (pH, ICP-OES). The concentration of Si^IV^ in the leachates showed the corrosive effects of each of the solutions, while the concentrations of Ca^2+^ and (PO_4_)^3–^ confirmed precipitation of a new Ca–P phase. The results confirmed earlier findings and showed that the TRIS and HEPES buffering systems are not inert and are strongly corrosive to bioglass 45S5. Moreover, their parts incorporate into the newly formed Ca–P layer and change its quality and crystallinity. These findings suggest that the current approach to *in vitro* testing of these materials requires reconsideration.

## Graphical abstract

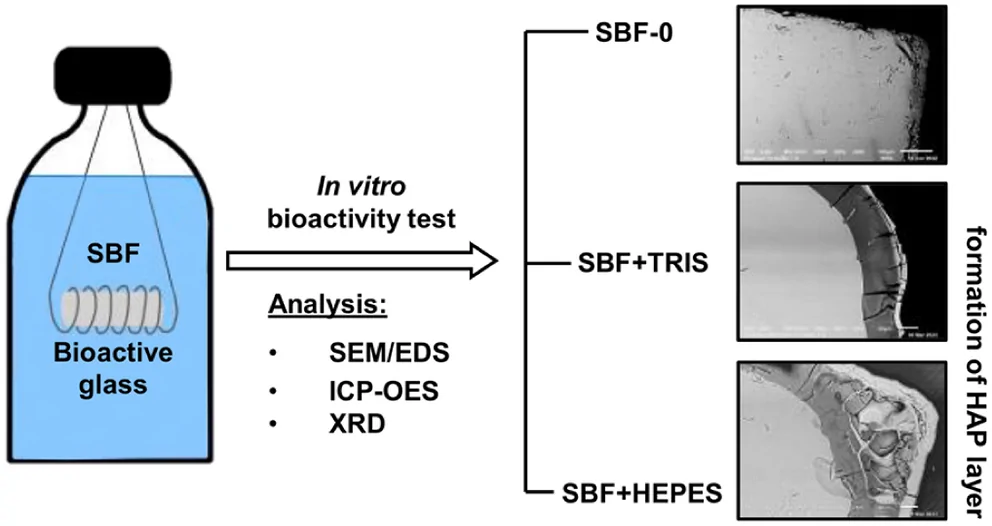

## 1 Introduction

The first step in testing bioactive materials for an “apatite forming ability” is to immerse them in an aqueous noncellular environment to study released ions and apatite precipitation.^1^ The simplest test solutions contain only inorganic components, such as saline (0.9% NaCl) or phosphate-buffered saline (PBS).^2^ Currently, the most commonly used method for *in vitro* bioactivity testing using simulated body fluid (SBF) is described in ISO standard 23317:2014 (recently updated as ISO 23317:2025).^3^ It is a solution similar in composition to the inorganic part of blood plasma; however, its composition is questionable. According to the ISO standard, the SBF solution has a five times lower concentration of HCO_3_^–^ ions and, on the contrary, too high concentration of chloride ions.^4^ The concentration of Cl^–^ ions produced is up to twice as high as that found in human plasma.

The increased concentration of Cl^–^ ions is not reflected in ISO standard 23317:2014. Another problem is the evaluation of the so-called apatite-forming ability. This ability (which can be simply described as bioactivity of the material) is evaluated in the ISO standard only based on the formation of hydroxyapatite (HAp). Another problem is the static design of the *in vitro* test. The standardized SBF solution is highly supersaturated toward apatite, the system is metastable, and the formation of HAp brings it to thermodynamic stability.^5^ Under the static conditions of the test, the Ca^2+^ and (PO_4_^3–^) ions are depleted after a few hours and HAp is no longer formed.^6^ The authors of the ISO standard believe that keeping the pH neutral during the *in vitro* test is crucial, as it affects the dissolution of glass/glass-ceramic materials.^7,8^ In human plasma, this function is performed by hydrocarbonates that are unstable under laboratory conditions.^9^ The pH stability in the SBF solution is maintained by the buffering system of TRIS (*tris­(hydroxymethyl)­aminomethane*) and HCl.^10^

In 1981, Irvin et al. were among the first scientists to demonstrate in a cell-based test that TRIS buffer is not completely physiologically inert and may interact with the surrounding system.^11^ Rohanová et al. demonstrated that TRIS buffer not only fails to maintain a stable pH but also actively interacts with glass-ceramic scaffolds. The TRIS/HCl buffer system accelerates dissolution of scaffolds and thus promotes formation of crystalline apatite (HAp) on their surface.^12^

A number of studies have been conducted to find a suitable alternative to TRIS buffer.^13–16^ Oyane et al. proposed three modified SBF solutions for *in vitro* testing. The ion composition of the r-SBF solution exactly matches the composition of blood plasma, m-SBF has a different concentration of HCO_3_^–^ ions, and i-SBF has a different concentration of Ca^2+^ ions. The pH stability in all these SBF solutions is maintained by using HEPES (*4-(2-hydroxyethyl)-1-piperazineethanesulfonic acid*) buffer.^13^ However, in another study by Rohanová et al., it has been proved that not even HEPES buffer is inert and it reacts with glass-ceramic scaffolds in a similar manner to TRIS buffer. HEPES buffer, like TRIS buffer, does not maintain a constant pH; it accelerates material dissolution and promotes formation of crystalline HAp.^14^ As alternatives to TRIS buffer, other buffers such as amino acids MOPS, BES and TES from the group of so-called Good’s buffers, have also been tested. Results of the studies showed that those buffers are not inert in SBF either and they as well as TRIS accelerate dissolution of the material. Moreover, the newly formed Ca–P phase has a different crystallinity (particle shape and size) in the presence of each of the buffers.^15,16^

As mentioned above, in human plasma, the buffer function is performed by hydrogen carbonates that are in equilibrium with the partial pressure of CO_2_. Unbuffered SBF solutions with HCO_3_^–^ ion concentrations closer to human plasma have also been tested for bioactivity.^17^ In these solutions, the dissolution of the glass-ceramic scaffolds is not as pronounced as in buffered solutions and only an amorphous layer of Ca–P forms on the surface. The tests have shown that with an increasing concentration of HCO_3_^–^ ions in unbuffered SBF, the solubility of the scaffolds slightly increased. Kulhánková et al. proposed an ideal concentration of 15 mmol·dm^–3^ of HCO_3_^–^ ions in unbuffered SBF, which is approximately half of the actual quantity of HCO_3_^–^ ions in blood plasma.^4^

Some authors have tried to replace the SBF solution with solutions primarily designed for cell culturing, such as DMEM solution (Dulbecco Modified Eagle Medium). DMEM is closer in composition to human plasma, but it has a more than 1.5 times higher concentration of HCO_3_^–^ ions (44 mmol·dm^–3^) and, conversely, a lower concentration of Ca^2+^ ions (only 1.8 mmol·dm^–3^). In addition to its inorganic part, it also contains organic matter.^18^ Furthermore, DMEM is enriched with growth factors, proteins, hormones, and vitamins contained in human plasma.^19^ Another solution that also includes glucose-enriched saline is known as Hank’s balanced solution (HBSS).^2^ However, not even these solutions fully meet the requirements for a balanced solution simulating blood plasma, and other alternatives must be sought.

The interaction of TRIS buffer with glass-ceramic scaffolds confirms that the evaluation of material reactivity according to the ISO standard, i.e., based only on HAp formation, provides only partial information about the reactivity of the biomaterial surface. Therefore, HAp formation should not be the only indicator when evaluating the bioactivity of a material. In order to understand the behavior of a biomaterial in SBF solution, it is necessary to monitor changes in pH, and especially changes in the concentrations of Ca^2+^ and PO_4_^3–^ ions, as well as the release of dissolution markers that are not involved in other interactions, such as silicates in the case of glasses or glass ceramics.^6^

In previous studies,^6,12,14^ TRIS and HEPES buffers were tested on glass-ceramic scaffolds, while in this work, we used the basic bioglass material 45S5 on which the changes caused by the presence of organic buffers can be observed much more easily. The objective of this work was to observe the corrosion behavior of TRIS and HEPES buffers on bulk samples of bioglass 45S5 and to compare it with the behavior in the unbuffered SBF solution. Bulk samples of bioglass 45S5 were tested in SBF+T solution prepared according to ISO 23317:2014 (neutralized with HCl), SBF+H (neutralized with NaOH), and SBF-0 (SBF without buffer). The *in vitro* testing was performed under static-dynamic conditions with daily solution exchange at 36.5 °C. Concentrations of Si^IV^, Ca^2+^, and PO_4_^3–^ ions were analyzed in the leachates by ICP-OES, and the surface of bioglass 45S5 was analyzed by SEM/EDS, XRD, and FTIR.

Our study follows the work^20^ in which bioglass 45S5 was tested in the form of crushed glass particles (0.3–0.5 mm) under static conditions. In contrast, in the present study, bioglass 45S5 was used in bulk form, which allowed for better observation and analysis of the quality of the newly formed layers as well as the measurement of their thickness. This approach allowed for a more systematic evaluation of buffer effects and a clearer distinction between material behavior and buffer influence. The results confirm previous findings obtained on glass-ceramic scaffolds and further indicate that the current approach to *in vitro* testing of the apatite formation ability of these bioactive materials requires reconsideration.

## 2 Experimental Section

### 2.1 Material Preparation and Analysis

The material tested was bioglass 45S5. The glass was melted in a crucible made of PtRh alloy at 1300 °C for 4 h in an electric laboratory furnace. To homogenize the glass, the mixture was stirred twice during the melting process. The glass was then cast onto a smooth steel mold and transferred to a cooling oven heated to 560 °C, where it was tempered for 1 h. After the tempering, it was cooled to laboratory temperature at a rate of 1 °C per minute.

Table 1 shows the composition of bioglass 45S5 prepared in our laboratory (measured by SEM/EDS). The impurity detected in the glass was mainly Al_2_O_3_. The mentioned oxide was introduced into the glass by the SiO_2_ raw material supplied by Lach-Ner, s.r.o. The bioglass 45S5 samples were ground with SiC grinding papers P120 and P400 into 8 × 6 × 2.5 mm bulk samples weighing 0.400 ± 0.100 g.

**Table 1:** Composition of Bioactive Glass Obtained by EDS Analysis [wt %]

oxide	SiO_2_	Na_2_O	CaO	P_2_O_5_	Al_2_O_3_	MgO
**wt %**	46.5	24.1	22.5	6.2	0.6	0.1

### 2.2 Solutions—Preparation

The bioglass 45S5 samples were exposed to three types of simulated body fluid solutions (Table 2). The solution marked **SBF+T** was prepared according to ISO standard 23317:2014 (Table 3). Hygroscopic components (K_2_HPO_4_·3H_2_O, MgCl_2_·6H_2_O, and CaCl_2_) were dried at 120 °C for 2 h before weighing.

**Table 2:** Ion Composition of SBF+T, SBF+H, SBF-0, and Human Blood Plasma (BP) in mmol·dm^–3^ ^3^

	Na^+^	K^+^	Ca^2+^	Mg^2+^	Cl^–^	HCO_3_^–^	HPO_4_^2–^	SO_4_^2–^	buffer
SBF+T	142	5	2.5	1.5	^a^178.0	4.2	1	0.5	50
SBF+H	142	5	2.5	1.5	^a^124.2	4.2	1	0.5	50
SBF-0	142	5	2.5	1.5	^a^149.3	4.2	1	0.5	-
BP	142	5	2.5	1.5	103	27	1	0.5	-

a The bold values of Cl^–^ ions were calculated and correspond to the actual concentration, considering the addition of HCl.

**Table 3:** Quantities of Reagents for the Preparation of 1000 mL of SBF+T (ISO Standard), SBF+H, and SBF-0

		Quantities [g]		
Order	Reagent	SBF+T	SBF+H	SBF-0
1	NaCl	8.035	6.598	8.035
2	NaHCO_3_	0.355	0.355	0.355
3	KCl	0.225	0.225	0.225
4	K_2_HPO_4_·3H_2_O	0.231	0.231	0.231
5	MgCl_2_·6H_2_O	0.331	0.331	0.331
6	CaCl_2_	0.292	0.292	0.292
7	Na_2_SO_4_	0.072	0.072	0.072
8	TRIS	6.118	-	-
9	HEPES	-	11.915	-
10	HCl (1M)	36 mL	-	0.25 mL
11	NaOH (1M)	-	22 mL	-

The raw material/component weights for the SBF+H and SBF-0 solutions were calculated so that the resulting ionic concentrations corresponded to the standard SBF solution. The **SBF+H** solution was prepared fresh weekly according to ISO standard 23317:2014 (Table 3). HEPES buffer is an amino acid, and therefore, the pH of the solution had to be increased with 1 M NaOH. This introduced Na^+^ ions into the solution, and the content of NaCl from the standard amount was taken into account. HEPES buffer was added before CaCl_2_ to avoid turbidity of the solution. With 22 mL of 1 M NaOH, the pH of the solution was adjusted so that the resulting solution had a pH = 7.4 at 36.5 °C. The **SBF-0** solution was also prepared fresh weekly according to ISO standard 23317:2014 (Table 3), except that TRIS buffer was not added to the solution and the pH was adjusted to 7.35 with 1 M HCl before adding CaCl_2_.

### 2.3 Static-Dynamic Conditions of In Vitro Tests

To prevent the effect of exhaustion of ions from the solutions, the so-called static-dynamic test was carried out in which, although the testing solutions did not flow continually around the sample as in dynamic tests, the solution was replaced on a daily basis (50 mL per day), i.e., every 24 h. Glass samples were placed in platinum spirals and submerged in 50 mL plastic bottles filled with either SBF+T, SBF+H, or SBF-0. The bottles with the samples were placed into a thermostat maintaining the temperature at 36.5 ± 0.5 °C. The interaction time was 14 days. At each selected time interval (after 1, 3, 7, 10, and 14 days), the exposure of two parallel samples was ended, and they were left to dry at laboratory temperature. Although drying caused some surface cracking, this did not affect the subsequent analyses.

### 2.4 Solutions (Leachates) Analysis

#### 2.4.1 ICP-OES

Elemental concentrations of Ca, P, and Si were determined by inductively coupled plasma emission spectrometry using a 5900 ICP-OES system (Agilent, USA). The plasma was generated from an Ar flow using a water-cooled semiconductor radiofrequency generator with a frequency of 27 Hz and power of 1.2 kW. The measurements were performed in a so-called synchronous vertical dual-view (SVDV) mode that allows measurements in axial and radial views simultaneously. The quantitative analysis was based on a six-point calibration series for each measured element. Each sample was measured with three repetitions. The signal intensities for Ca were measured at wavelength λ = 396.847 nm, for P at λ = 213.618 nm, and for Si at λ = 288.158 nm. The measured values were evaluated using ICP Expert Pro software.

#### 2.4.2 pH Measurement

The pH measurements were performed using an inoLab pH meter with a combined glass electrode around 35 °C, due to the natural cooling of the sample after removal from the thermostat.

### 2.5 Material Analysis

#### 2.5.1 SEM/EDS

Scanning electron microscopy (SEM) was used to observe the glass morphology and to identify chemical compositions of the glasses (EDS). The samples were embedded in epoxy resin in a perpendicular position, ground (with SiC papers P120, P600, and P1200) in ethanol, and then polished with a diamond paste (1 μm) on a textile wheel. The analyses were performed with a JEOL JSM 6510 electron microscope equipped with an INCA EDS SSD detector (Oxford Instruments) in a backscattered secondary electron (BSE) mode, providing chemical contrast. Measurements were performed at an accelerating voltage of 20 kV under low pressure (30 Pa). Each measurement is an average of three analyses. The data obtained were normalized to 100%. Three-component glasses were prepared from p.a.-purity chemicals K_2_O-CaO-SiO_2_ and Na_2_O-CaO-SiO_2_ in a molar ratio of 75 mol % SiO_2_, 15 mol % CaO, and 10 mol % Na_2_O or K_2_O. The chemical composition of the glass was checked by XRF. The glass was melted from Sigma-Aldrich chemicals and high-purity glass sand. Standards (59 metal/mineral combinations) from a certified laboratory (*SPI Supplies brand*) were used. The measurements were carried out at the Institute of Inorganic Chemistry of the Czech Academy of Sciences, Husinec-Řež.

#### 2.5.2 FTIR

Attenuated total reflectance (ATR) Fourier-transform infrared (FTIR) spectra were obtained using a Thermo Nicolet Nexus 670 FTIR spectrometer equipped with a single-reflection SMART-iTX ATR accessory and MCT-A liquid nitrogen-cooled detector for high sensitivity with a spectral range from 11,700 cm^–1^ to 660 cm^–1^. The samples of bioglass 45S5 were submerged for 10 days in SBF solutions. Then, the bioglass 45S5 samples’ surface was scratched to increase the sample’s area in contact with the ATR crystal, and the obtained powders were pressed onto the ATR diamond surface. The analyses were performed by Jan Labuta, Ph.D., at the Research Center for Materials Nanoarchitectonics (MANA-NIMS), Tsukuba, Japan.

#### 2.5.3 X-ray Powder Diffraction—Conventional Bragg–Brentano Reflection Geometry

Diffraction patterns were collected with a PANalytical X́Pert PRO diffractometer equipped with a conventional X-ray tube (Cu K_α_ radiation, 40 kV, 30 mA) and a linear position-sensitive detector PIXcel with an antiscatter shield. A programmable divergence slit set to the fixed value of 0.25 deg., Soller slit of 0.04 rad, and mask of 5 mm were used in the primary beam. A programmable antiscatter slit set to the fixed value of 0.25 deg., Soller slit of 0.04 rad, and Ni beta filter were used in the diffracted beam. Data were collected in the range of 5–85 deg. 2theta with a step of 0.0131 deg. and 500 s/step producing a scan of about 3 h 35 min. A qualitative analysis was performed with a HighScorePlus software package (Malvern PANalytical, The Netherlands, version 5.2.0)^21^ together with the PDF-5+ database.^22^

## 3 Results

### 3.1 Analysis of Leachates

The values of **pH** increased on the first day of interaction in all the solutions; however, it increased only to 7.5 in the buffered SBF+T and SBF+H solutions while in the SBF-0 solution, it increased to 8.2 (Figure 1a). Changes in silicon (**Si** ^**IV**^) concentrations reflected dissolution of the bioglass 45S5 material. Figure 1b clearly shows that in the buffered solutions, although the pH did not change significantly, the glass dissolved strongly.

**Figure 1:**
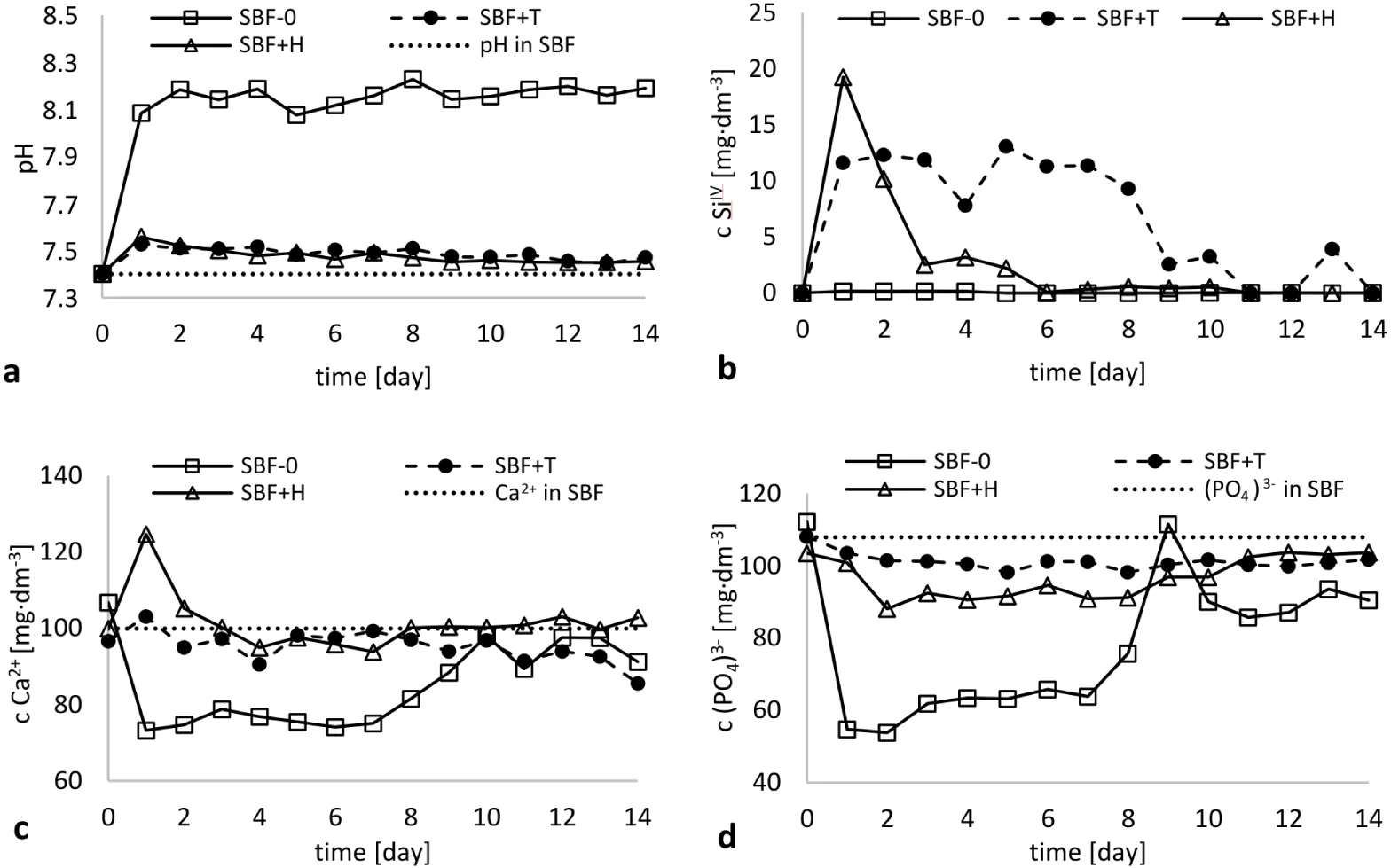
pH values (a) in SBF-0, SBF+T, and SBF+H with 45S5 glass; (b) concentration of Si^IV^ in SBF-0, SBF+T, and SBF+H with 45S5 glass; (c) concentration of Ca^2+^ ions in SBF-0, SBF+T, and SBF+H with 45S5 glass; (d) concentration of PO_4_^3–^ ions in SBF-0, SBF+T, and SBF+H with 45S5 glass.

As early as on the first day, 12–19 mg·dm^–3^ **Si** ^**IV**^ was released into the leachate, representing about 1 wt % of its total weight. In the SBF+H solution, the dissolution of the material slowed down after the first day and stopped completely after the sixth day. In the TRIS-buffered solution (SBF+T), the dissolution continued until the eighth day and then it stopped as well. The dissolution of bioglass 45S5 in buffered solutions is also obvious from the small increase in **Ca** ^**2+**^ ion concentration on the first day of exposure (Figure 1c). In the following days, the Ca^2+^ concentration stabilized around 80–90 mg·dm^–3^ in all the solutions. From the beginning of the interaction, **PO**_**4**_^**3–**^ ions were removed from the glass into all of the solutions to **form a Ca–P** layer (Figure 1d).

### 3.2 Analysis of Materials

Monitoring the **weight** changes of the bioglass 45S5 samples after the interaction with the above-mentioned solutions confirmed that the greatest dissolution occurred in the buffered solutions SBF+T and SBF+H (Figure 2a).

**Figure 2:**
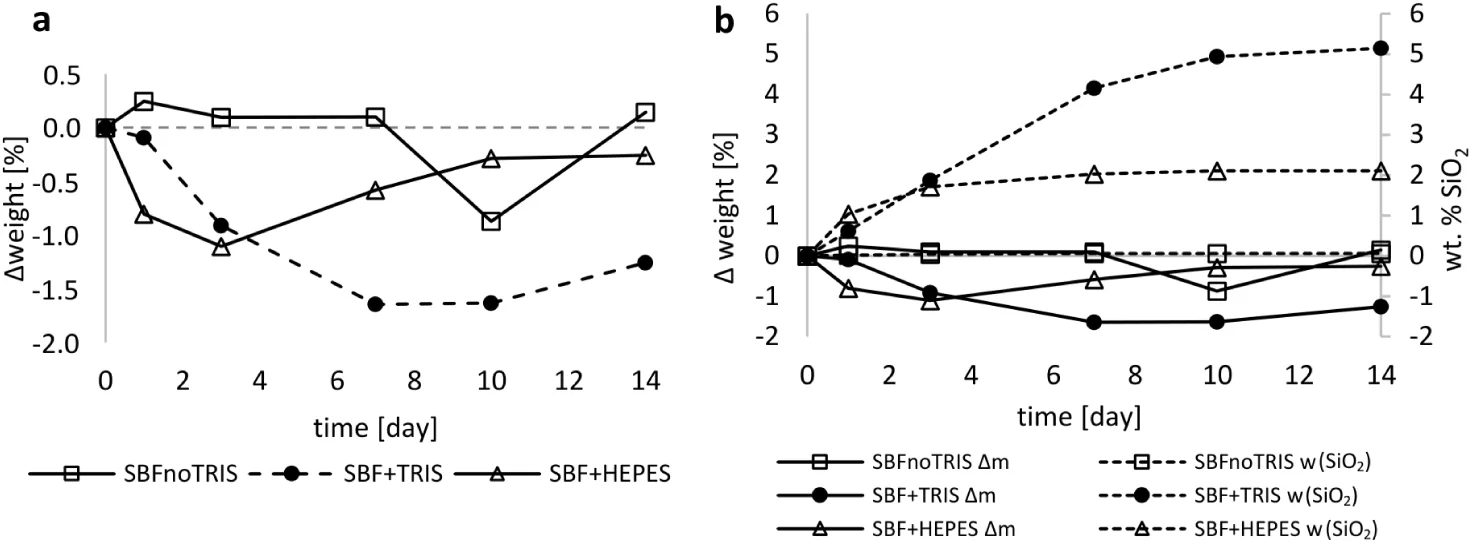
Weight changes of 45S5 glass (a) in SBF-0, SBF+T, and SBF+H; (b) cumulative dissolution of SiO_2_ in SBF-0, SBF+T, and SBF+H, as calculated from the material balance of leached Si^IV^.

On the basis of the mass balance of Si (the difference between w_Si_ in the original material and w_Si_ leached into the solution) and assuming that silicon did not precipitate back into the newly formed layer, the **amount of the newly formed** Ca–P phase was calculated. The diagram (Figure 2b) shows that the biggest amount of Ca–P precipitate was formed in the SBF+T solution (up to ca. 30 mg), up to three times less precipitate was formed in the SBF+H solution (ca. 10–13 mg), and only about 1–3 mg of the new Ca–P phase was formed in the SBF-0 solution.

**XRD** analysis of the surface **layers** confirmed the formation of **hydroxyapatite** (HAp) in the buffered solutions after only 1 day of *in vitro* testing. In addition to HAp, NaCl was also formed on the surface, which has been discussed previously.^18^ In the SBF-0 solution, besides crystalline NaCl, no other crystalline phases were detected. The presence of OCP, as a precursor of HAp^23^ [p. 394, Figure 8], was not detected (Figure 3).

**Figure 3:**
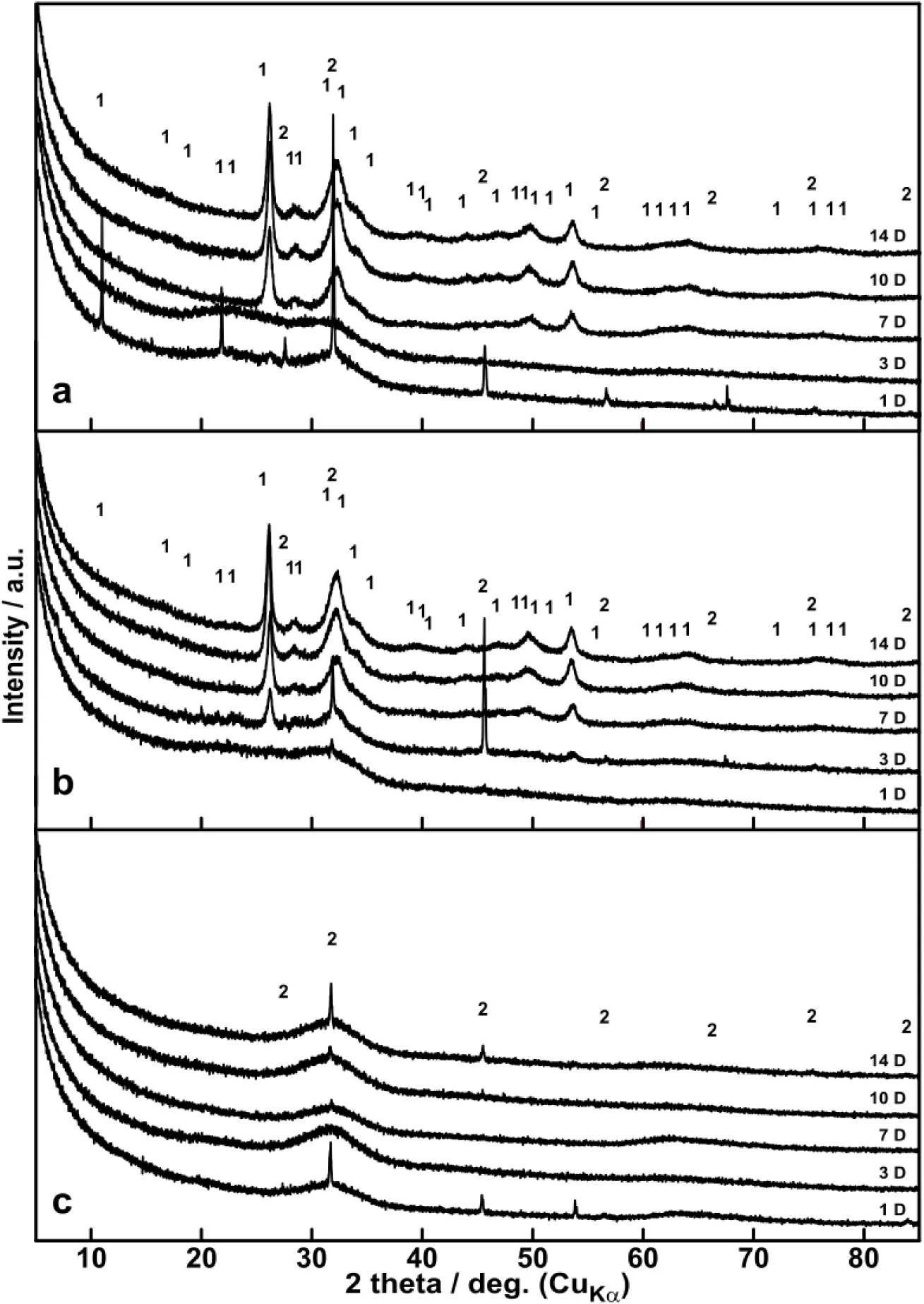
XRD patterns of 45S5 glass with (a) SBF+T after 14 days, (b) SBF+H after 14 days, and (c) SBF-0 after 14 days.

The recorded FTIR spectra in Figure 4 show the formation of HAp only on the surface of bioglass 45S5 samples submerged in the buffered SBF solutions (Figure 4, red and blue lines). A strong band around 1023 cm^–1^, including bands at 1090 and 962 cm^–1^, is characteristic of the hydroxyapatite of the PO_4_^3–^ group^24–26^ (see the control FTIR spectrum of pure HAp powder, gray line) and is present in both buffered samples (red and blue spectra), whereas bioglass 45S5 in nonbuffered SBF (SBF-0) lacks these absorption bands (green spectrum). Moreover, a small carbonate CO_3_^2–^ substitution in HAp^27,28^ is present in both SBF buffered samples due to uptake from the solution during the HAp formation.^29^ Similarly, there is a broad band from 3600 to 2750 cm^–1^ corresponding to water uptake during HAp formation,^24^ which is present only in the buffered samples. There was no evidence of HAp formation on the surface of bioglass 45S5 in the nonbuffered SBF solution (Figure 4, green spectrum). It is worth mentioning that a closer distinction of a minor admixture (e.g., the presence of SO_4_^2–^ groups) was not possible due to the admixture’s low concentration and low ATR-FTIR sensitivity.

**Figure 4:**
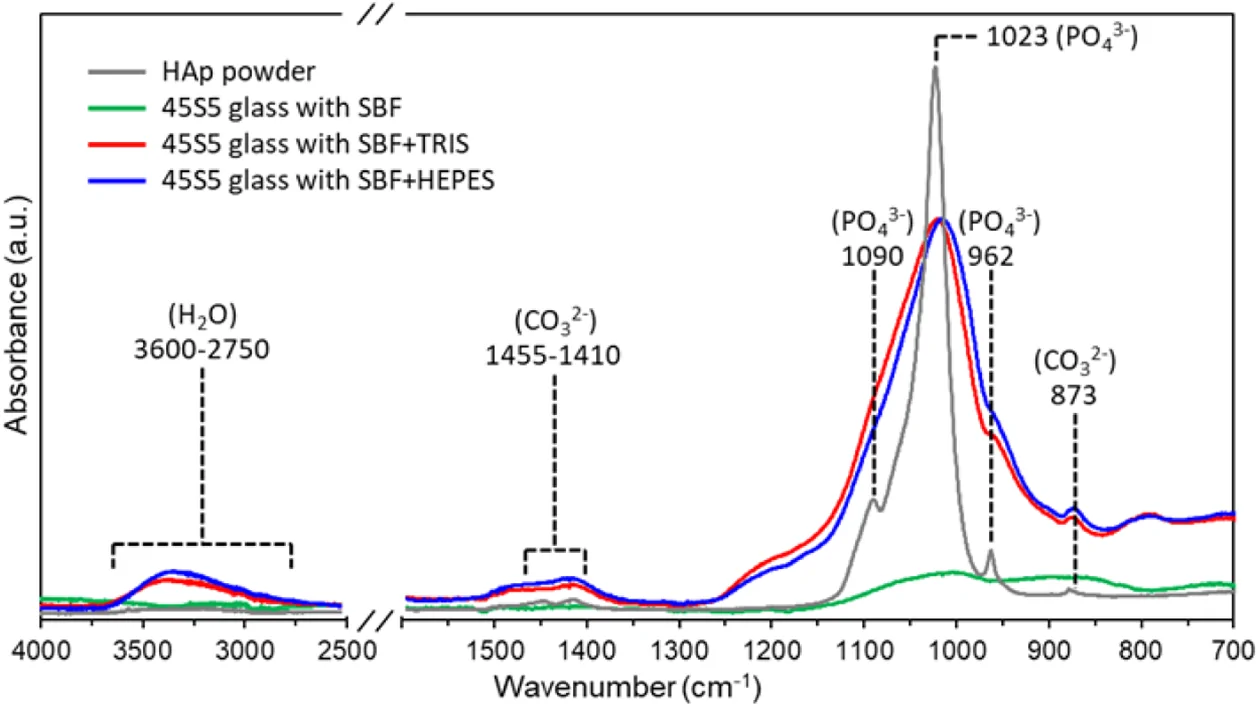
ATR-FTIR spectra (at room temperature) of a pure HAp powder control sample (gray spectrum and powders obtained from surface scratches of 45S5 bioglass which were submerged for 10 days in nonbuffered SBF (green spectrum), in TRIS-buffered SBF (red spectrum), and in HEPES-buffered SBF (blue spectrum).

Monitoring the **thickness of the layers** and **their composition** (by SEM/EDS, Figure 5) after *in vitro* testing in the buffered solutions allowed us to distinguish the corroded layer containing mainly SiO_2_ from the newly formed Ca–P layer (Figure 5a and b). Between the two layers, there is a part that is “transitional”, formed by both SiO_2_ and calcium and phosphorus. Table 4 presents the results of the chemical analysis of those layers (EDS), and it is interesting to observe in detail their composition over time. For example, in the SBF+T solution, chlorine was incorporated into the layer after the first day of exposure and after the tenth day, the layer contained about 3 wt % Cl^–^ ions. In the SBF+H solution, SO_4_^2–^ ions were incorporated into the layers, they were observed in close proximity to the still-intact glass, and by the end of the test (tenth day), they represented up to 15 wt % (Table 4). In the SBF-0 solution, the glass surface was not corroded even after the 14 day test. Formation of a very thin calcium phosphate layer, amorphous in nature, was observed on the surface (Figure 5c and d).

**Table 4:** Composition of Newly Formed Layers Obtained by EDS Analysis [wt.%]

Oxide/	SiO_2_ corroded layer					SiO_2_ + Ca–P interlayer					HAp layer				
Day	1	3	7	10	14	1	3	7	10	14	1	3	7	10	14
**SBF+T**															
**SiO**_**2**_	58.3	73.3	82.1	78.5	85.1	-	58.7	77.3	37.9	6.2	44.4	6	7	8.4	4.2
**P**_**2**_ **O**_**5**_	16.8	10.3	4.7	7	5.2	-	17.9	7.3	27.5	40.3	23.3	39.7	39.4	39.9	41.4
**CaO**	16.9	10.7	6	8.3	5.7	-	16.5	8.1	25.5	45.3	22.5	43.4	43.3	42.2	44.9
**Na**_**2**_ **O**	3.8	3.3	3.9	3.3	1.8	-	3.1	3.3	2.1	3	3.7	4.9	5	4	3.4
**MgO**	1.5	0.9	0.4	0.6	0.3	-	1	0.4	1.9	1.5	2.1	1.6	1.7	2.1	1.8
**Al**_**2**_ **O**_**3**_	1.2	1.1	1.3	1.2	1.3	-	1.2	1.7	1	0.2	1.4	0.2	0.1	0.4	0.1
**SO**_**3**_	0.3	0	0	0	0	-	0.2	0	0	0.1	0.5	0.5	0.3	0.1	0.2
**K**_**2**_ **O**	0	0.2	0.2	0.3	0.2	-	16.5	0.2	0.3	0.1	0.2	0.1	0.1	0.1	0.1
**Cl**	1.1	0.2	0.8	0.5	0.3	-	0.2	0.9	2.4	2.2	1.5	2.3	2	3	2.6
**SBF+H**															
**SiO**_**2**_	27.6	53.4	63.9	36.6	82.4	-	25.4	25.5	56.8	33.3	11.4	6.4	4.1	3.8	3
**P**_**2**_ **O**_**5**_	21.3	17.1	15.2	10.3	4.1	-	30.9	34.7	16.7	22.7	31.2	40.9	44.9	42.6	44.2
**CaO**	19.8	16.9	15.6	21.4	5.2	-	29.7	32	16.6	29.3	40.7	40.7	45.2	42	43
**Na**_**2**_ **O**	6.8	5.3	2.6	9.5	3.3	-	4.8	3.2	3.4	3.2	4.5	5.4	2.8	4.8	0
**MgO**	2.1	1.3	1.1	1.4	0.4	-	2.5	2.6	1.4	1.3	1.7	2.1	1.9	2.1	2.2
**Al**_**2**_ **O**_**3**_	1.4	1	1.1	0.5	1	-	1	1.3	1.2	1.7	0.7	0.1	0	0.1	0
**SO**_**3**_	8.9	3.2	0.3	15.1	2.3	-	3.2	0.3	2.6	2	5.1	2.3	0.6	1.9	1.1
**K**_**2**_ **O**	0.4	0.2	0.1	0	0.3	-	0.2	0	0.2	0.2	0.3	0.1	0	0.2	0.1

**Figure 5:**
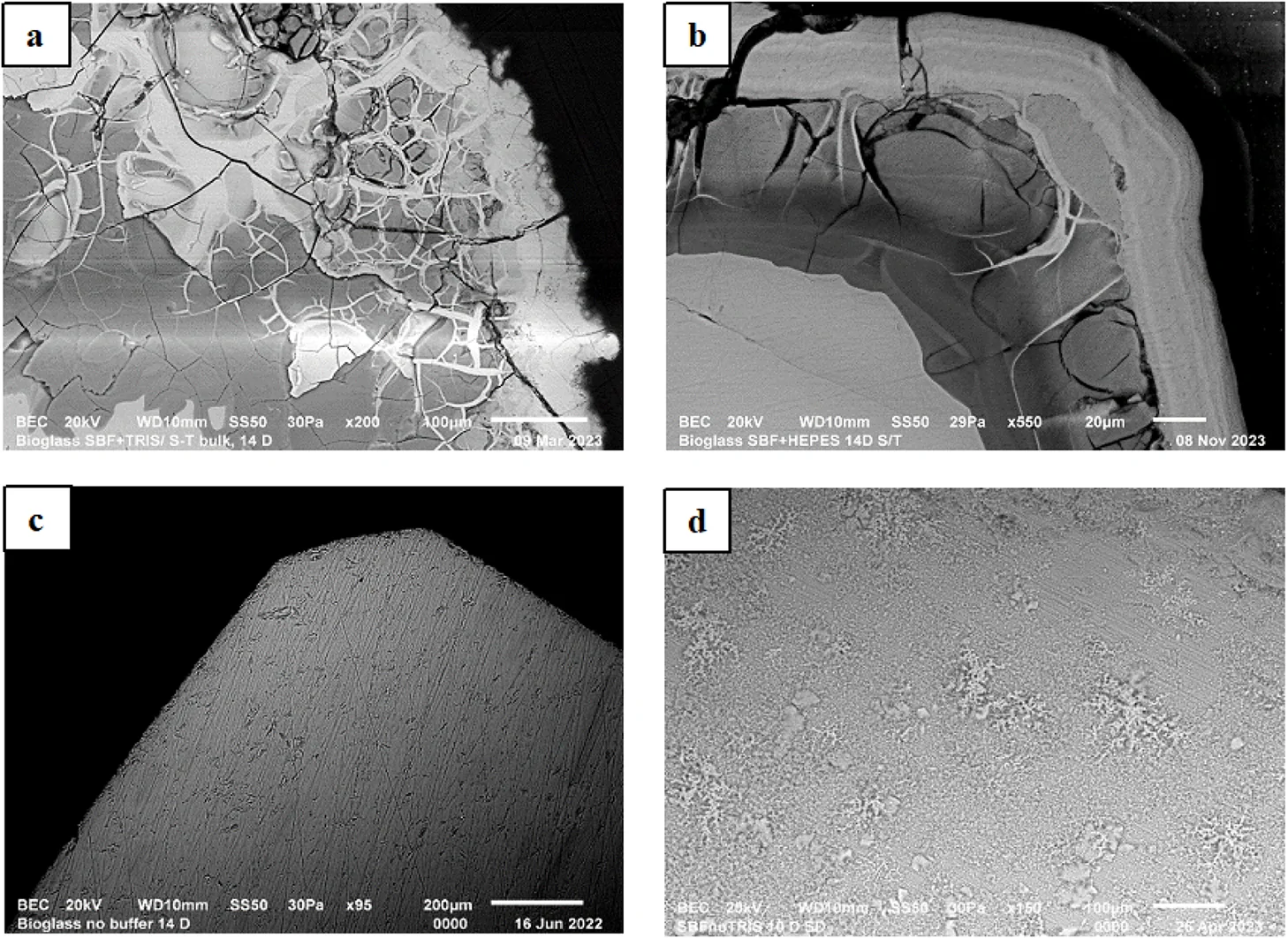
SEM images of bioactive glass samples after exposure: (a) SBF+T (day 14, perpendicular cut), (b) SBF+H (day 14, perpendicular cut), (c) SBF-0 (day 14, perpendicular cut), and (d) surface of 45S5 glass in SBF-0 (day 14).

## 4 Discussion

For years, we have been pointing out the discrepancies in the evaluation of *in vitro* tests according to ISO standard 23317:2014 (updated in 2025) where the organic salt TRIS (*tris­(hydroxymethyl)­aminomethane*) neutralized with HCl is used to maintain a neutral pH value. Initially, we assumed the formation of soluble complexes of TRIS buffer with Ca^2+^ ions that could inhibit deposition of the apatite layer on the material surface, thus reducing its protective effect.^30^ However, later, it was proved that the TRIS + HCl system actively interacts with the tested bioglass/glass ceramics and acts as a significant corrosive agent. In an attempt to replace it, we tested amino acids from a series of the so-called Good’s buffers (HEPES, BES, TES, and MOPS) which were neutralized with NaOH.

However, a strong corrosive effect on glass-ceramic materials was shown for all buffer systems tested.^14,15^ In our previous study,^16^ where we focused on glass-ceramic scaffolds prepared from bioglass 45S5, we pointed out the fact that the buffer not only interacts with the material but also affects the quality and crystallinity of the newly formed apatite layer. In this work, for simplicity, we have used bulk samples of the 45S5 base bioglass and demonstrated the effects of organic buffers or rather buffer systems. Both the tested buffers had a strong corrosive effect on the glass, and the corrosion occurred at different rates (Figure 6). It is also interesting to look closely at the composition of the corroded surfaces of bioglass 45S5 and the thicknesses of the newly formed layers in the buffered solutions (Figure 7a and b). The corroded and newly formed layer in SBF+T was up to approximately 150 μm-thick after 14 days of exposure. Of this, 110 μm is the corroded layer (the corrosion rate was approximately 8 μm·day^–1^). The apatite layer is about 40 μm-thick (Figure 7a) which corresponds to a growth rate of 3 μm·day^–1^.

**Figure 6:**
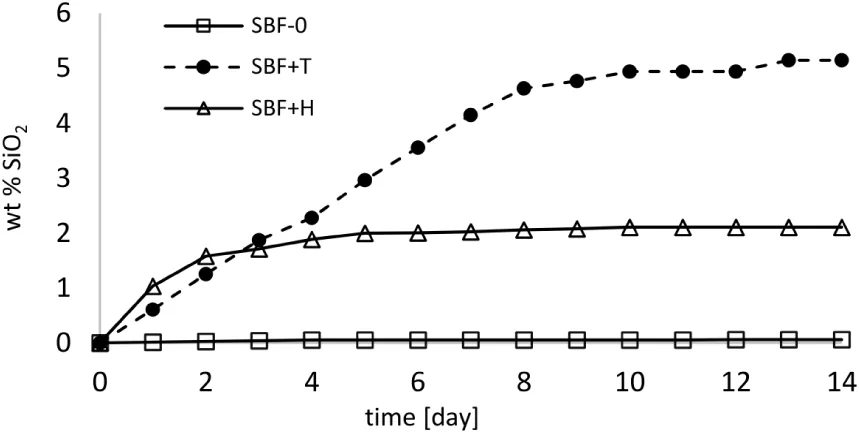
Cumulative dissolution of released SiO_2_ from 45S5 glass in SBF-0, SBF+T, and SBF+H solutions.

**Figure 7:**
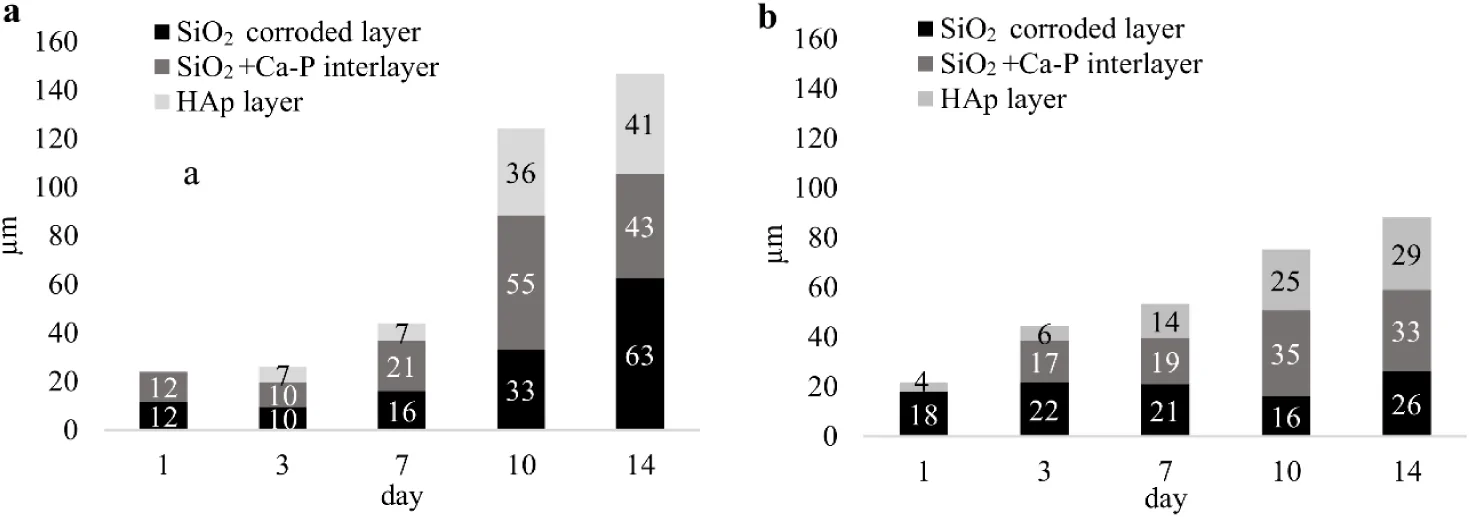
Thicknesses of the corroded and apatite layers formed in (a) SBF+T and (b) SBF+H.

The corrosion behavior of the TRIS buffer solution itself (0.05 M TRIS + 0.04 M HCl) under static conditions was also tested. A thick corroded layer (approximately 20–40 μm) containing mainly SiO_2_ was formed on the surface of bioglass 45S5. Moreover, a thin hydroxyapatite layer (around 10 μm) was formed from the leached bioglass 45S5 components (Ca and P).^31^ These results are in accordance with the behavior of the TRIS + HCl buffering system tested on glass-ceramic scaffolds.^12^

In the SBF+H solution, a layer of similar composition was formed, but the thickness of the corroded SiO_2_ layer did not change after the first day and remained approximately 20 μm-thick until the end of the experiment (after 14 days) (Figure 7b), representing a corrosion rate of around 1.4 μm·day^–1^. The hydroxyapatite layer was approximately 25 μm-thick (1.5 μm·day^–1^), which is about half of that formed in SBF+T. From the facts described above, it is clear that TRIS buffer corrodes bioglass 45S5 more intensely and the thicknesses and growth rates of the newly precipitated layers correspond to this.

From the values of accumulated SiO_2_, it is obvious that in the SBF-0 solution, the glass almost did not dissolve (Figure 6).

How can we explain this phenomenon? The appearance of the bioglass 45S5 surface exposed to the buffered solutions (Figure 8a and b) may provide a clue. The layer formed on bioglass 45S5 in SBF+T is heavily cracked and easily peeled off, but it remains permeable for the solution. EDS chemical analysis has shown that it contains chloride ions, and XRD confirmed the presence of NaCl[Fn fn1]. In contrast, the HAp layer formed after exposure to SBF+H is very dense and shows no signs of flaking, and after four to 5 days of exposure, it is impermeable to the SBF solution.

**Figure 8:**
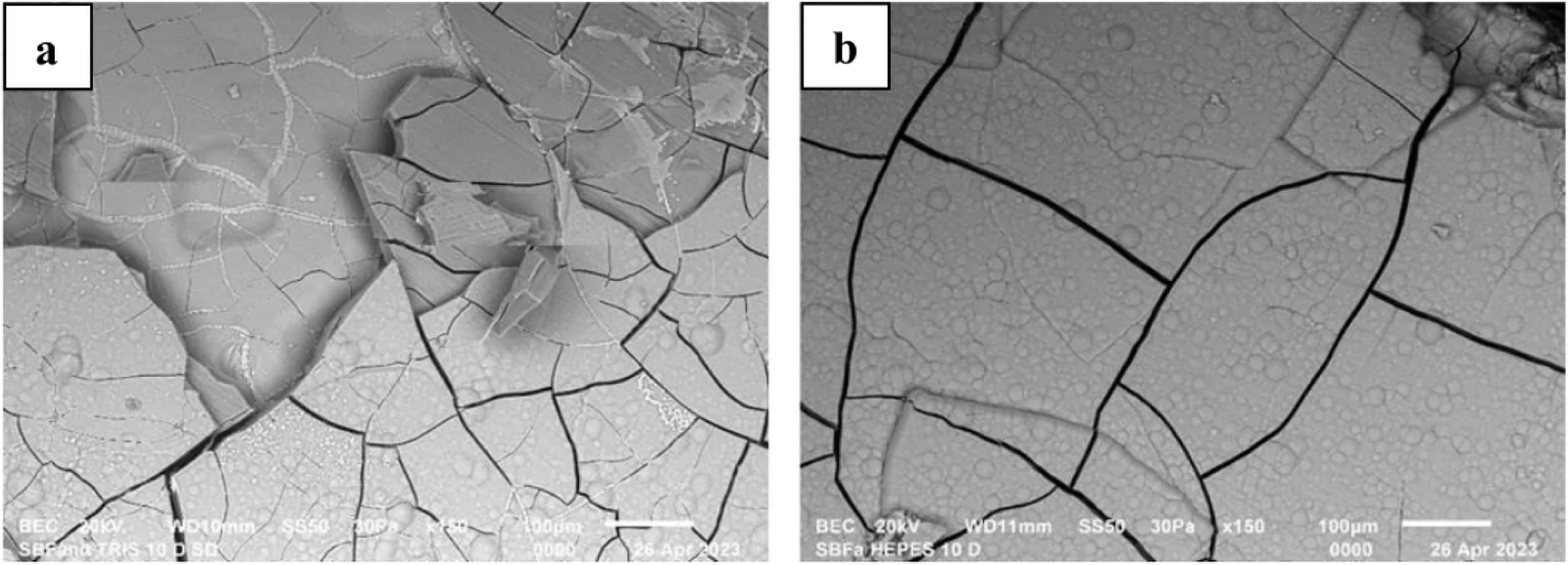
Morphology of the bioglass 45S5 surface exposed to (a) SBF+T and (b) SBF+H.

In the layer containing mainly SiO_2_ and in the corroded interlayer, we have detected sulfate SO_4_^2–^ ions that come from the HEPES buffer itself and change place with PO_4_^3–^ ions, as described by Siddiqi and Azhar^32^ [p. 152, Chapter 6.2.2, Table 6.1].

Each of the tested buffering systems supported the formation of a HAp layer of completely different quality. This finding is very surprising, and again, it confirms that the **buffering systems actively interact with the tested material** and have a major influence on the morphology, composition, thickness, and adhesion of the newly formed apatite layer. For example, the corrosion of bioglass 45S5 in buffered environments involves several rapid, simultaneous processes. The TRIS buffer, most probably, introduces hydroxide ions (OH^–^) into the solution, which readily disrupt the [SiO_4_]^4–^ network, resulting in the formation of soluble Si­(OH)_4_ species^33^ [p. 58–65]. The kinetics of this initial silica dissolution significantly influence subsequent phase transformations, including the precipitation of calcium phosphate. One notable process is the diffusion of Na^+^ ions to the glass surface, where they immediately react with Cl^–^ ions, introduced in excess during the TRIS neutralization with HCl. This reaction leads to alkali depletion and the formation of a highly permeable, ion-exchangeable corrosion layer as well as the precipitation of NaCl. This layer facilitates the transport of ionic species both from the glass and the surrounding simulated body fluid (SBF). The localized enrichment of Ca^2+^ at the glass surface, combined with the metastable nature of SBF, promotes rapid nucleation and growth of hydroxyapatite (HAp). As the HAp layer thickens, it progressively slows further glass corrosion, eventually reaching a quasi-steady state within several days. However, these conditions are not representative of the physiological environment. Our findings demonstrate that the formation of HAp in these systems is primarily induced by the buffering agents themselves. The second studied buffering system (HEPES + NaOH) similarly increases the concentration of (OH)^−^ ions during neutralization, accelerating both glass corrosion and mineralization. On the contrary, unbuffered SBF (SBF-0), representing the inorganic plasma fraction, causes negligible glass corrosion. This yields results that poorly reflect *in vivo* conditions and may lead to false-negative assessments of bioactivity. The dissolution kinetics of bioglass in plasma are critical for the *in situ* formation of apatite layers.

## 5 Conclusion

This experiment has demonstrated that the organic buffering systems (in this case, TRIS + HCl and HEPES + NaOH) used to maintain a neutral environment in the SBF solution when testing glass, glass-ceramic, and ceramic materials promote the formation of hydroxyapatite and thus generate false-positive results of *in vitro* tests. It is misleading and insufficient to deem the formation of hydroxyapatite on the surface of materials with potential bioactive capabilities as the only proof of their bioactive behavior. In the unbuffered solution, no crystalline apatite was formed but only amorphous calcium phosphate. Consequently, not even the unbuffered solution fully reflects reality, and it is not completely suitable for the testing.

It is necessary to examine not only the surface of the bioglass but also the concentrations of ions in the leachates after exposure. Therefore, a proper understanding of the *in vitro* test results can be achieved only by combining the two approaches. It is essential to reconsider not only the use of organic buffer systems but also the entire procedure of *in vitro* testing of bioglass currently conducted according to ISO standard 23317:2014 (updated in 2025).

## Footnotes

1 NaCl crystallized from the SBF+TRIS/HCl solution, and it was not caused by insufficient rinsing of the sample after exposure to SBF, as we proved in a previous study.
